# Lean body mass wasting and toxicity in early breast cancer patients receiving anthracyclines

**DOI:** 10.18632/oncotarget.25394

**Published:** 2018-05-22

**Authors:** Federica Mazzuca, Concetta Elisa Onesti, Michela Roberto, Marco Di Girolamo, Andrea Botticelli, Paola Begini, Lidia Strigari, Paolo Marchetti, Maurizio Muscaritoli

**Affiliations:** ^1^ Department of Clinical and Molecular Medicine, “Sapienza” University of Rome, Rome, Italy; ^2^ Department of Medical Oncology, Sant’Andrea Hospital, Rome, Italy; ^3^ Department of Medical Oncology, University Hospital (CHU) and University of Liège, Liège, Belgium; ^4^ Department of Radiology, Sant’Andrea Hospital, Rome, Italy; ^5^ Department of Gastroenterology, Sant’Andrea Hospital, Rome, Italy; ^6^ Laboratory of Medical Physics and Expert Systems, Regina Elena National Cancer Institute, Rome, Italy; ^7^ Department of Clinical Medicine, Sapienza University of Rome, Rome, Italy

**Keywords:** breast cancer, sarcopenia, lean body mass, anthracyclines toxicity, adjuvant chemotherapy

## Abstract

**Background:**

Sarcopenia refers to the reduction of both volume and number of skeletal muscle fibers. Lean body mass loss is associated with survival, quality of life and tolerance to treatment in cancer patients. The aim of our study is to analyse the association between toxicities and sarcopenia in early breast cancer patients receiving adjuvant treatment.

**Materials and Methods:**

Breast cancer patients who have received anthracycline-based adjuvant treatment were retrospectively enrolled. CT scan images performed before, during and after adjuvant chemotherapy were used to evaluate lean body mass at third lumbar vertebra level with the software Slice Omatic V 5.0.

**Results:**

21 stage I–III breast cancer patients were enrolled. According to the skeletal muscle index at third lumbar vertebra cut-off ≤38.5 cm^2^/m^2^, 8 patients (38.1%) were classified as sarcopenic before starting treatment, while 10 patients (47.6%) were sarcopenic at the end of treatment. A lower baseline L3 skeletal muscle index is associated with G3-4 vs G0-2 toxicities (33.4 cm^2^/m^2^ (31.1–39.9) *vs* 40.5 cm^2^/m^2^ (33.4–52.0), *p* = 0.028). Similarly skeletal muscle cross sectional area was significantly lower in patients with G3-4 toxicities (86.7 cm^2^ (82.6–104.7) *vs* 109.0 cm^2^ (83.3–143.9), *p* = 0.017). L3 skeletal muscle index is an independent predictor of severe toxicity (*p* = 0.0282) in multivariate analysis.

**Conclusion:**

Lean body mass loss is associated with higher grade of toxicity in early breast cancer patients receiving adjuvant chemotherapy.

## INTRODUCTION

Sarcopenia, historically referred to the age-related reduction of both volume and number of muscle fibers. It leads to a functional capacity impairment with decreased strength, metabolic rate and aerobic capacity [1, 2].

Sarcopenia shows a multifactorial origin including disuse, endocrine function alterations, chronic diseases, inflammation and nutritional deficiencies [3]. Contrary to cachexia, sarcopenia may occur without a concomitant loss of fat mass and with a various percentage of lean body mass reduction.

Lean body mass (LBM) is the predominant source of organism proteins which are essentials for any cellular process. Moreover, a loss of LBM and an excessive protein catabolism, as it happens in cancer patients, may lead to a decline in immune activity with increased adverse clinical outcomes [4]. Protein synthesis dysregulation may be responsible for sarcopenia. Indeed, an inadequate response to anabolic stimuli or the upregulation of proteolysis in cancer are the principle causes of muscles loss [4]. In several studies, it has been showed that a lower LBM is associated with decreased survival, quality of life and tolerance to treatment [5]. The study of both fat and LBM at the level of the third lumbar vertebra (L3), performed by dual-energy X-ray absorptiometry (DXA) and/or computed tomography (CT) scan, appears to be strongly predictive of the entire body composition [6–10]. Indeed, CT scan provide details on each inspected muscle, adipose tissue and organs, representing an objective and reproducible technique for sarcopenia evaluation as well as cancer stage assessment [7].

The exact prevalence of sarcopenia is currently unknown, due to the variety of definitions proposed, the various techniques used to detect it and the different frequencies described in several diseases [11–13]. However, a careful estimation of sarcopenia and changes in body composition could represent an important way to prevent chemotherapy toxicity and to improve quality of life and survival of cancer patients.

In this retrospective analysis, we studied the association between sarcopenia and toxicity in early breast cancer patients receiving adjuvant treatment.

## RESULTS

Twenty-one stage I–III breast cancer patients who have received anthracycline-based adjuvant treatment at the Department of Medical Oncology, Sant’Andrea Hospital of Rome between February 2013 and April 2014 were retrospectively enrolled. The median age was 54 years (range: 39–72 years). Tumor histology, HER-2 and hormonal status as well as more detailed information about surgery and chemotherapy are shown in Table 1. The median body composition parameters, i.e Body Mass Index (BMI), Body Surface Area (BSA), L3 Skeletal Muscle Index (L3 SMI) and Skeletal Muscle Cross Sectional Area (CSA) at baseline and after 4 cycles of chemotherapy are reported in Table 2. Using the cut off ≤ 38.5 cm^2^/m^2^ for L3 SMI to define sarcopenia, 8 patients (38.1%) were sarcopenic before starting treatment and 10 patients (47.6%) after 4 cycles of chemotherapy, with a 9.5% increase in frequency during treatment [11]. Only 2 patients (9.5%) were obese (BMI 31.2 and 33.7 respectively), but not sarcopenic. A proportion of 33.3% of the patients were overweight, 14.2% of witch sarcopenic according to ≤38.5 cm^2^/m^2^ cut-off.

**Table 1:** Basal characteristic of the 21 enrolled patients

	Number of patients	%
**Age (years)**		
**Median 54 (39–72)**		
**Histology**		
**Ductal**	18	85.7
**Lobular**	2	9.5
**Undifferentiated**	1	4.7
**Stage**		
**IA**	7	33.3
**IIA**	5	23.8
**IIB**	6	28.6
**IIIA**	3	14.3
**ER**		
**Positive**	17	81
**negative**	4	19
**PgR**		
**Positive**	16	76.2
**Negative**	5	23.8
**HER-2**		
**Amplified**	9	42.9
**Not amplified**	12	57.1
**Surgery**		
**Mastectomy**	9	42.9
**Quadrantectomy**	12	57.1
**Chemotherapy**		
**FEC**	5	23.8
**ET**	2	9.5
**EC/AC^à^ Ptx/Txt + Trastuzumab**	9	42.9
**EC^à^ Ptx/Txt**	5	23.8

Abbreviations: FEC: 5-fluorouracil-Epirubicin-Cyclophosphamide; ET: Epirubicin-Docetaxel; EC: Epirubicin-Cyclophosphamide; AC: Adraimicin-Cyclophosphamide; Ptx: Paclitaxel; Txt: Docetaxel.

**Table 2:** BMI, BSA and lean body mass index for the 21 enrolled patients before and after treatment

	Before treatment (T0)		After treatment (T2)	
	Median	Range	Median	Range
**BMI (kg/cm**^**2**^)	23.6	18.0–33.7	23.2	17.2–33.7
**BSA (m^2^)**	1.7	1.4–1.9	1.7	1.4–2.1
**L3 SMI (cm^2^/m^2^)**	39.2	31.6–52.9	39.2	31.6–52.9
**skeletal muscle cross-sectional area (cm^2^)**	103.3	82.6–143.9	104.5	83.2–135.5
**Weigth (Kg)**	63.5	46.0–82.0	60	44–95
**Height (cm)**	161	150–175	161	150–175

Overall, we observed G3-4 toxicity in 19% of the cases, mainly neutropenia (9.5%), vomit (4.8%), diarrhea (4.8%), mucositis (4.8%) and abdominal pain (4.8%), as showed in Table 3.

**Table 3:** Toxicities reported during treatment for the 21 enrolled patients

	G1-2 toxicity		G3-4 toxicity	
	*N*	%	*N*	%
**Neutropenia**	6	28.6	2	9.5
**Anemia**	11	52.4	0	0
**Nausea**	16	76.2	0	0
**Vomit**	7	33.3	1	4.8
**Diarrhea**	6	28.6	1	4.8
**Constipation**	3	14.3	0	0
**Dysgeusia**	9	42.9	0	0
**Mucositis**	5	23.8	1	4.8
**Abdominal pain**	2	9.5	1	4.8
**Neurotoxicity**	10	47.6	0	0
**HFS**	15	71.4	0	0
**Cutaneous**	4	19.0	0	0
**Asthenia**	17	81.0	0	0
**Alopecia**	21	100	0	0
**Insomnia**	5	23.8	0	0
**Depression**	2	9.5	0	0
**Ocular**	1	4.8	0	0
**Fever**	1	4.8	0	0

**Figure 1:**
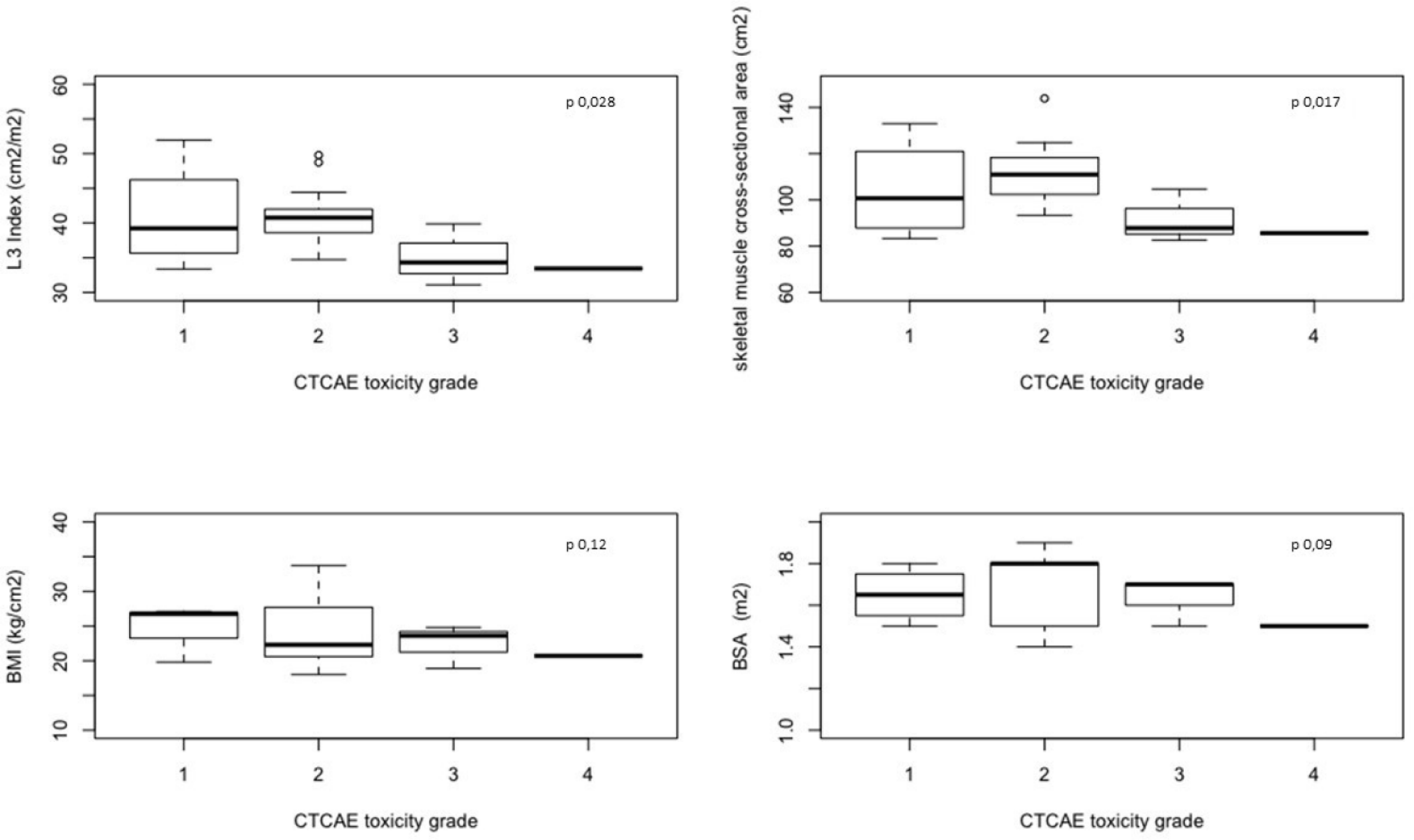
L3 Index, skeletal muscle cross-sectional area, BMI and BSA basal value in the 21 enrolled patients according to toxicity grade

**Figure 2:**
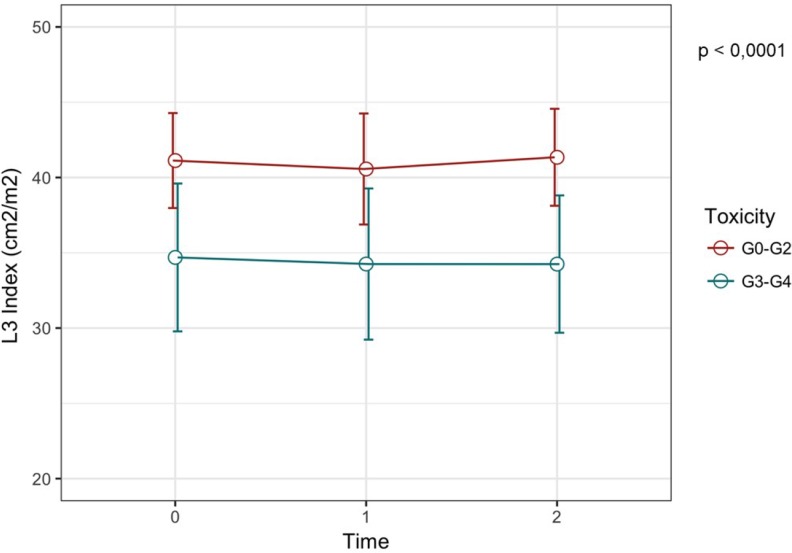
L3 Index variation over time according to toxicity grade The red line show the L3 SMI at T0, T1 and T2 for the group of patients with G0-2 toxicities, while the blue line represent the variation of L3 SMI for patients with severe toxicities. We can observe that patients with G3-4 toxicities have a lower L3 SMI.

**Figure 3:**
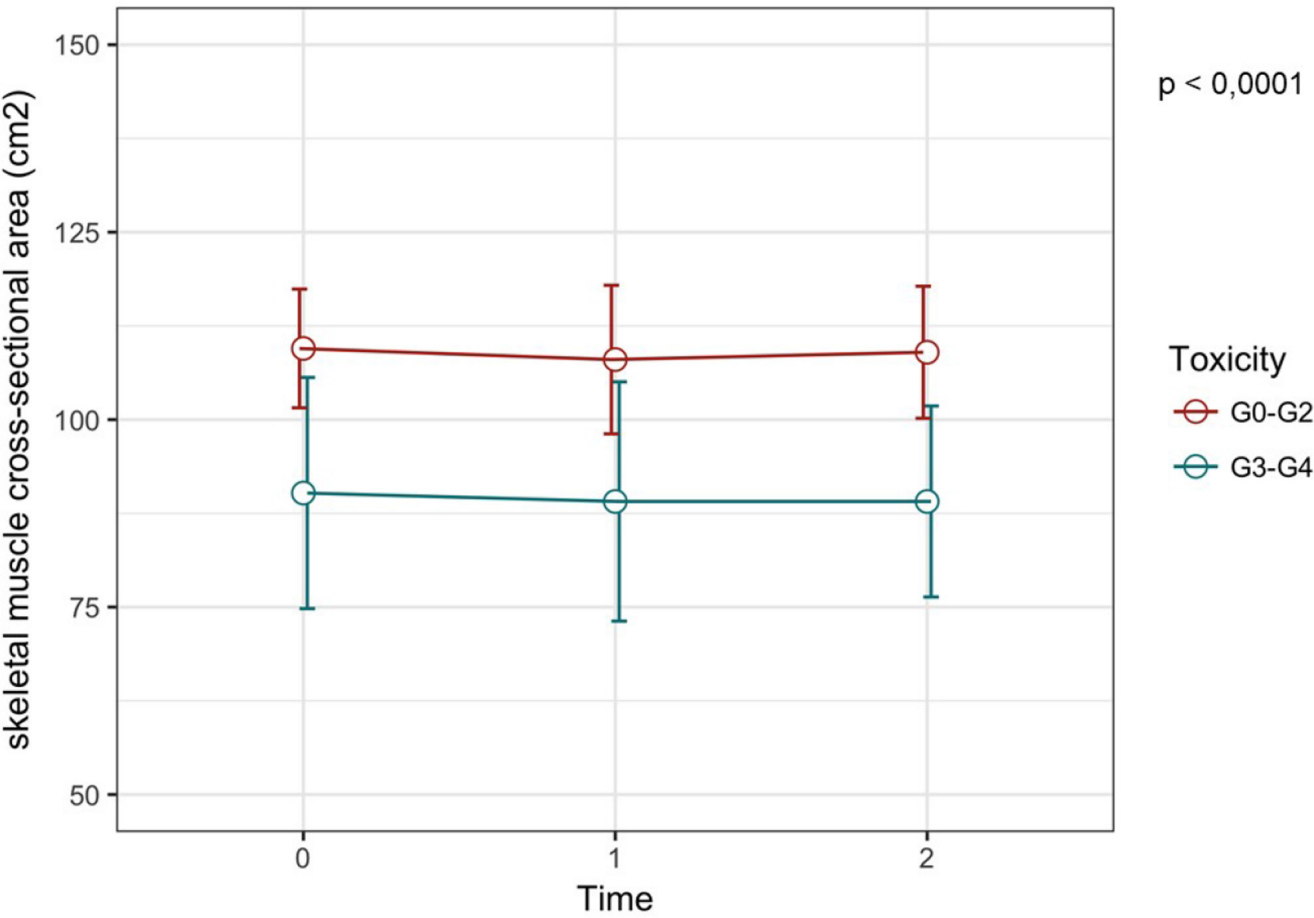
Skeletal muscle cross-sectional area variation according to toxicity grade The red line show the skeletal muscle CSA at T0, T1 and T2 for the group of patients with G0-2 toxicities, while the blue line represent the variation of skeletal muscle CSA for patients with severe toxicities. We can observe that patients with G3-4 toxicities have a lower skeletal muscle CSA.

The baseline value of L3 SMI has been shown to be significantly lower in patients with G3-4 toxicities compared to patients with G0-2 toxicities (33.4 cm^2^/m^2^ (31.1–39.9) *vs* 40.5 cm^2^/m^2^ (33.4–52.0) respectively, *p* = 0.028). Moreover, also the skeletal muscle CSA was significantly lower in G3-4 compared to patients with G0-2 toxicities (86.7 cm^2^ (82.6–104.7) *vs* 109.0 cm^2^ (83.3–143.9) respectively, *p* = 0.017). Meanwhile, BSA and BMI values were not associated with toxicities (Table 4 and Figure 1). A lower value of both L3 SMI (Figure 2, *p*-value < 0.0001) and skeletal muscle CSA (Figure 3, *p*-value < 0.0001) has been showed to be significantly correlated with G3-4 toxicity also during (Time 1 in Figures 2 and 3) and after 4 cycles (Time 2 in Figures 2 and 3) of chemotherapy.

**Table 4:** Basal BMI, BSA, L3 index and skeletal muscle cross-sectional area according to absent/mild and severe CTC toxicity

	Absent/mild toxicity (i.e. ≤G2)	Sever toxicity (≥G3)	*p*-value
**BMI (kg/cm^2^)**	26.4 (range: 18–33.7)	22.2 (range: 18.9–24.8)	0.12
**BSA (m^2^)**	1.7 (range: 1.4–1.9)	1.5 (range:1.4–1.7)	0.09
**L3 SMI (cm^2^/m^2^)**	40.5 (range: 33.4–52.0)	33.4 (range: 31.1–39.9)	0.028
**skeletal muscle cross-sectional area (cm^2^)**	109.0 (range: 83.3–143.9)	86.7 (range: 82.6–104.7)	0.017

Finally, a multivariate analysis adjusted for basal BMI, BSA, L3 SMI and CSA revealed that L3 SMI was an independent predictor of severe toxicity (*p* = 0.0282).

## DISCUSSION

Sarcopenia is a common condition in cancer patients, due to altered protein catabolism [4]. Different instrumental techniques have been used to study body composition [6–10]. However, the evaluation of adipose tissue and LBM at the level of the L3 vertebra, performed by DXA and/or CT scan, appears to be the best choice [11, 13].

The European Working Group on sarcopenia recommends to evaluuate the presence of both low muscle mass and low muscle function (strength or performance) to determine sarcopenia [14]. However, in our study we analysed only the loss of muscle mass. In fact, being the study retrospective, it was not possible to collect informations about muscle function.

Based on the hypothesis that body composition is relevant in drugs’ distribution and activity, we studied the association between sarcopenia and toxicities in breast cancer patients receiving adjuvant epirubicin-based treatment.

Several studies showed that the decrease of LBM is associated with higher chemotherapy toxicities, as well as a worse outcome in various cancer type [5, 15–40]. Moreover, a muscle wasting without adipose tissue and body weight reduction was observed during anticancer treatment with both chemotherapy and target therapy [25, 41]. Focusing on studies conducted on breast cancer, Prado and colleagues published a study performed on 55 metastatic patients treated with capecitabine, evaluating muscle mass by CT scans at L3 vertebra level. About 25% of patients were identified as sarcopenic and 50% of them showed grade 2 or greater toxicities. Only 20% of non-sarcopenic patients had severe toxicities. They observed also a shorter time to progression (TTP) in sarcopenic patients [33]. Prado and colleagues conducted also a study on 24 breast cancer patients receiving adjuvant treatment, observing a higher LBM in patients without toxicity (*p* 0.002) and an association between epirubicin cleareance and LBM (*p* 0.041) [34]. Recently, Shachar and colleagues showed a significant association between sarcopenia and toxicities in metastatic breast cancer patients treated with taxanes (57% vs 18% of G3-4 toxicity in sarcopenic and non-sarcopenic group respectively, *p* 0.02) and in early breast cancer receiving anthracyclines and taxanes-based chemotherapy (G3-4 toxicity relative risk (RR) = 1.29 for low skeletal muscle index (SMI), *p* 0.002; RR= 1.09 for low skeletal muscle gauge (SMG), *p* 0.01) [35, 36]. Rier and colleagues observed an association between low muscle attenuation (LMA) and shorter OS in 166 metastatic breast cancers receiving first line chemotherapy [37]. Deluche and colleagues identified an association of L3 SMI with both DFS and OS in 119 breast cancer patients [38]. Finally, Villaseñor and colleagues observed that sarcopenia is associated with an increased risk of overall mortality in a cohort of 471 stage I–IIIA breast cancer patients [40].

Currently, the adjustment of anticancer drugs doses is based on physical parameters (weight, height and BSA), not considering changes in the relationship between fat and lean mass, typical of natural history of cancer patients [42]. Nevertheless, there are growing evidences that LBM can be a better parameter for the evaluation of chemotherapy doses. For instance, Gusella and colleagues demonstrated the wide correlation of the reduction in fat-free mass with the decrease in distribution volume and clearance of 5-fluorouracil [43]. Muscle tissue, in fact, in addition to acts in drugs’ metabolism, is a large area of drugs’ distribution, being richly vascularized. So, when there is a muscle reduction, an increase in drugs’ plasma concentration occurs., This is usually associated with higher grade of toxicities [13].

A large part of our cohort is sarcopenic at diagnosis. This observation leads to consider that the main responsibles of muscle loss are tumor-related factors or pro-inflammatory cytokines, instead of chemotherapy related factors. Another hypotesis is that patients with diagnosis of breast cancer reduce their physical activity due to the decrease of the mood.

In line with literature data we found that sarcopenic status was significantly associated to higher risk of developing toxicities. Moreover, we observes that treatment induces a raise in sarcopenia frequency in about 9.5% of the cases. This is probably due to the reduced food intake or to the reduced physical activity consequent to anticancer treatment.

Even though the small sample size and the retrospectivity of the analysis, these findings suggest the importance to consider body composition in determining drugs’ doses. The prevention of LBM impairment could be useful to improve the quality of life of patients receiving chemotherapy.

During recent years, a growing interest in muscle mass maintenance in cancer patients has been observed. Most appetite stimulants or nutritional agents have been widely studied for prevention of cancer cachexia, leading to an increase in fat without an increase in lean body mass. Anabolic androgenic steroids showed more successful results in treating skeletal muscle wasting with an increased protein synthesis. The use of androgenic steroids is associated with several adverse events, such as hepatic toxicity, acne, increased sebum production, virilization and hirsutism in women. Moreover, there is a potential relation with prostate cancer and benign prostatic hyperplasia in men [44, 45]. Selective androgen receptor modulators (SARMs) are currently studied. The advantages of this class of drugs is that they can selectively induce anabolism without the accompanying androgenic side effects. Dobs and colleagues showed, in a recently completed phase II trial, a significant increase in lean body mass in patients treated with enobosarm than in patients treated with placebo [46]. Moreover, two phase III trials of enobosarm to treat sarcopenia in metastatic NSCLC undergoing first line therapy have been recently completed (POWER trials; https://clinicaltrials.gov NCT01355484 and NCT01355497), showing a gain in lean body mass in the experimental arm [47].

In conclusion, our results support literature data showing a relation between sarcopenia and toxicity in cancer patients. In fact, sarcopenia is associated to worse outcome, to higher risk of complication after surgical intervention, to higher mortality and to the risk of developing severe toxicities [5, 13]. In particular, adverse events are the major cause of dose reduction, delaying of administration and therapy discontinuation. Dose intensity loss avoidance has to be considered as a primary purpose of cancer treatment, above all in adjuvant setting. Furthermore, management of severe toxicities are a cause of health costs raise and quality of life impairment.

Future clinical trials investigating dose assessment based on LBM status, along with studies on sarcopenia prevention, have the potential to personalize treatment and to improve outcome and quality of life during chemotherapy.

## MATERIALS AND METHODS

### Patients selection

Stage I–III breast cancer patients who have received anthracycline-based adjuvant treatment at the Department of Medical Oncology, Sant’Andrea Hospital of Rome between February 2013 and April 2014 were retrospectively analysed.

The eligibility criteria was: histological diagnosis of early breast cancer, adjuvant anthracycline-based adjuvant chemotherapy received for at least 4 cycles, CT scan available before (Time 0, T0), during (Time 1, T1) and after (Time 2, T2) adjuvant chemotherapy.

BMI, BSA and toxicity according to the National Cancer Institute Common Toxicity Criteria (CTCAE) version 4.0 were assessed at each chemotherapy cycles.

The primary end-point of this study is to assess the association between sarcopenia, expressed as L3 SMI and skeletal muscle CSA, and toxicity in early breast cancer patient receiving adjuvant anthracycline. The secondary end-points are to assess the prevalence of sarcopenia in early breast cancer patients and to explore the variation of lean body mass during chemotherapy.

All the patients signed an informed consent. The study was conducted in accordance with the Declaration of Helsinki and the institutional ethic committee approved the protocols.

### Image analysis for sarcopenia

Sarcopenia was evaluated by the analysis of available CT scan images performed before (T0), during (T1) and after (T2) adjuvant chemotherapy. A transverse section with a multiplanar reconstruction at L3 was used to study the following muscles: psoas, paraspinal, abdominal transverse rectum, internal and external obliques. Images were analysed by the software Slice Omatic V 5.0 (Montreal, Quebec, Canada Tomovision). The muscles areas were marked by the Hounsfield unit thresholds from −29 to + 150 and the total CSA were computed. The CSA obtained in this way are normalized according to patients’ height. Finally, the L3 SMI was calculated. According to previous published studies, sarcopenia was defined by a L3 SMI value ≤38.5 cm^2^/m^2^, because it is independent from BMI. Considering that obese or overweight patients could be sarcopenic, this cut-off is more useful to recognize sarcopenia, as showed by Prado and colleagues [11].

### Statistics

The data were given as mean ± SD or median (range) as appropriate. Boxplots of patient-related parameters over time were used to show variability among groups. Differences between groups were calculated by using a two-tailed *t*-test. Linear regression analyses were used to estimate correlations between toxicity-reported and patient-related body composition data, carried-out before starting treatment, after the 2th and 4th cycle of chemotherapy. For the multivariate statistics, a stepwise forward multiple linear regression was performed. Differences were considered significant at *p*-value < 0.05. All statistics were calculated using R-Package (version 3.3.2).
